# Insights from a qualitative study of the procurement and manufacture of active pharmaceutical ingredients in India

**DOI:** 10.1136/bmjgh-2022-011588

**Published:** 2023-05-17

**Authors:** Heather Hamill, Kate Hampshire, Harshada Vinaya, Pavan Mamidi

**Affiliations:** 1 Department of Sociology, University of Oxford, Oxford, UK; 2 Department of Anthropology, Durham University, Durham, UK; 3 Department of Cognitive Science, University of California, La Jolla, San Diego, USA; 4 Centre for Social and Behavioural Change, Ashoka University, Delhi, India

**Keywords:** Qualitative study, Health economics, Health systems, Health policy

## Abstract

Medicine supply systems are a crucial part of health systems and access to effective essential medicines is a key pillar of Universal Health Coverage. However, efforts to expand access are compromised by the proliferation of substandard and falsified medicines. The vast majority of research to date on medicine supply chains has focused on the formulation and distribution of the finished product, overlooking the crucial steps of Active Pharmaceutical Ingredient production that precede this. In this paper, we draw on qualitative interviews with manufacturers and regulators in India to take a ‘deep dive’ into these understudied parts of medicine supply chains.

**Summary box**

- Poor-quality medicines are a major threat to healthcare provision in low-income and middle-income countries. Research on medicine supply chains highlights their complexity and opaqueness but focuses mostly on the formulation and distribution of the finished product.
- This study focuses on the procurement of raw materials and the manufacture of Active Pharmaceutical Ingredient. We note the attrition rate among manufacturing companies, cost pressures and weak and variable regulatory oversight that may lead to minimal compliance with the overall effect of producing poorer quality products.
- There is a need to pay increased attention to the risks of regulatory circumvention or minimal compliance in the initial parts of medicine supply chains. Greater regulatory oversight and harmonisation is required to counteract sectorial myopia and keep pace with the globalisation and fragmentation of supply chains.

## Introduction

Medicine supply systems are a crucial part of health systems and access to effective essential medicines is a key pillar of Universal Health Coverage.^1^ However, efforts to expand access are compromised by the proliferation of poor-quality substandard or falsified (SF) medicines.^2–5^ According to the WHO, ‘substandard medicines result from errors, corruption, negligence or poor practice in manufacturing, procurement, regulation, transportation or storage. By contrast, falsified products result from criminal fraud.’ They estimate that over 10% of pharmaceuticals consumed in low-income and middle-income countries are poor quality.^6^

The structural causes of the problem of poor-quality medicines are well documented. Resource-poor populations and high disease burdens create a ready market for cheap medicines, while under-resourced regulatory agencies and weak judicial systems provide little incentive for suppliers to prioritise quality over profits.^7^ These ‘hard’ economic facts have confounded efforts by national/international agencies to curb the problem through tightening regulation, improving detection rates and public education campaigns.^8 9^ We also contend that existing polices have been limited by a failure to comprehend fully the complex workings of medicine supply systems from production to administration.

Medicine supply chains are noteworthy because of their complexity.^10 11^ In this paper, we discuss parts of the supply chain which have received relatively little scholarly attention: the procurement of raw materials and the manufacture of Active Pharmaceutical Ingredient (APIs) that are used to formulate branded or generic pharmaceuticals. We hope to contribute to a better understanding of how poor-quality medicines enter supply chains by taking a ‘deep dive’ into the complexity of the earlier stages of the manufacturing process and noting key weaknesses in regulatory requirements.

## Background: medicine regulation, API manufacture and gaps in current research

Medicine regulations have been defined as the ‘combination of legal, administrative and technical measures that governments take to ensure the safety, efficacy and quality of medicines, as well as the relevance and accuracy of product information.’^12^ Regulatory compliance is generally understood in the scholarly and policy literature as ‘obedience by a target population with regulations’.^13^ For compliance to occur three key conditions need to be met. First, the target group must be aware of the rule and understand it. Second, the target group must be willing to comply either because of economic incentives, positive attitudes arising from a sense of good citizenship, acceptance of policy goals or pressure from enforcement activities. And third, the target group must be able to comply. Much of the work on compliance has centred on understanding the barriers to these three conditions being met and thus the barriers to compliance.^14^ However, compliance with rules does not always ensure that regulation is effective in achieving its goals. In this paper, we suggest that the complexity and opacity of transnational pharmaceutical supply chains may lead to the quality of the final product being compromised, despite regulatory compliance being achieved at each stage.

The paper focuses on APIs defined as ‘any substance or mixture of substances intended to be used in the manufacture of a drug (medicinal) product and that, when used in the production of a drug, becomes an active ingredient of the drug product. Such substances are intended to furnish pharmacological activity or other direct effect in the diagnosis, cure, mitigation, treatment or prevention of disease or to affect the structure and function of the body.’^15^ The manufacture of APIs from raw ingredients entails several intermediate stages, which often takes place in different production facilities, moving between countries in the process.^16^ The finished API is then combined with excipients, chemically inactive substances that help the medication remain stable and control absorption when the medicine is administered.^17^ Both APIs and excipients are crucial to the formation of an efficacious finished product. Most research to date has focused on the latter stages of medicine production: the combination of API and excipients (and packaging) to create the finished product, thereby overlooking the complexities and potential vulnerabilities that occur further upstream. It is this important gap that the current paper seeks to address. In contrast, the API-step is a crucial issue for regulators and the WHO Prequalification of Medicines Programme, which aims to ensure that medicines supplied by procurement agencies meet acceptable standards of quality, safety and efficacy covers both finished pharmaceutical products and APIs.^18 19^

### Data collection from manufacturers and regulators in India

Our findings draw on research conducted as part of a study on ‘Trust, risk and uncertainty in pharmaceutical transactions in sub-Saharan Africa’ that focuses on how actors in pharmaceutical supply chains come to trust medicines and therapeutic techniques under conditions of uncertainty about medicine quality. As India is the largest exporter of pharmaceuticals to the African continent, we followed the supply chain to include manufacturers there. In 2019, three of the authors together conducted eight interviews in Delhi and Hyderabad, India with manufactures (N=6) and regulators (N=2) of pharmaceutical products. The interviewees were sampled using a snowball sampling strategy. A trusted industry insider introduced the researchers, and the study aims to the interviewees.

The six manufacturing businesses varied in terms of size of company, type of product manufactured (API, intermediate, formulation) and whether it was for export or for the Indian market only. The interviewees were either Chief Executive Officers (CEOs) responsible for the overall management and operation of the company, or in senior management positions within their companies and thus able to offer both strategic and operational insight. The two regulators held senior leadership positions at agencies responsible for pharmaceutical manufacture regulation at the regional level.

The interviews were semistructured and conversational in tone and style. Each interview began with an explanation of the research aims, how the data would be used, and verbal consent was given. The interviews with manufacturers included questions about manufacturing processes, transportation, quality control and quality assurance and the risks and vulnerabilities across manufacturing and distribution supply chains. The interviews with regulators included questions about the agencies’ regulatory responsibilities, individual roles and the daily challenges individuals faced in fulfilling their statutory duties. All identities of the informants and the companies and organisations they work for remain anonymous throughout this paper.

The sample size is small, but as Hennick and Kaiser note, this does not mean it is inadequate.^20^ The sample’s homogeneity, the narrow and in-depth focus of the interview questions and the information-rich participants meant that data saturation was reached on the topics of interest.

Interviewers took hand-written notes rather than audio recordings, because of the potential sensitivity of the material. The authors typed up their notes and shared them with one another to enhance the reliability of the data. The interviews were analysed following the principles of grounded theory whereby theoretical insight emerges from data through an iterative process of close-reading and coding.^21^ In the first cycle, data were coded descriptively. In the second cycle, descriptive codes were collapsed into more analytical themes that provide insight into key processes and vulnerabilities in the manufacturing, quality control and quality assurance processes. The quotations throughout this paper are illustrative of experiences reported by the wider sample.

## Author reflexivity statement

See online supplemental appendix 1.^22^

10.1136/bmjgh-2022-011588.supp1 Supplementary data

### Patient and public involvement statement

Patients or the public were not involved in the design, or conduct, or reporting, or dissemination plans of our research.

## The Indian pharmaceutical industry and API supply chains

The Indian pharmaceutical industry is the world’s third largest by volume and 14th largest in terms of value.^23^ It is the largest supplier of generic medicines globally with an estimated 3000 drug companies and 10 500 manufacturing units^24^ gaining it the informal title of ‘Pharmacy to the World’, something regarded by our interviewees as a source of national pride.

Our interviewees confirmed the importance of ensuring quality at the earliest stages of the manufacturing process. One, for example, was adamant that ‘It is the consistent quality of the raw materials that gives a good quality API, and consistent quality of the API that results in a consistent quality end product’ (Interview, A). In contrast to much of the published literature on SF medicines, which focuses on the final formulation (the branded or generic product), one of our interviewees noted that, ‘If there are six steps in making a medicine, most steps are in the production of the API and the last five or six steps are in the formulation of the final product’ (Interview, B) making a clear case for increased scrutiny of these earlier stages of the production process.

The sheer complexity of API supply chains was highlighted by all our interviewees. For example, 1 of the companies in our sample has over 600 suppliers of raw materials used in the manufacture of intermediates and APIs, of which approximately 280 were in China, 250 in India, 60–70 in Europe and about 10 in the USA (Interview A). In many cases, APIs are not manufactured in a single plant: some companies we interviewed would import raw materials from China and complete the first step of API manufacturing before exporting the intermediate (often back to China) for further processing, after which they will return to India for the final stage of manufacture into an API. Thus, API production involves the transportation of raw materials and intermediates across large distances, passing multiple borders, jurisdictions and regulatory agencies. Such long and fragmented supply chains are a major challenge for quality control and quality assurance and heavily reliant on regulations being adhered to by multiple and dispersed actors.

## The quality assurance and quality control

Interviewee A leads a team of 15 people (13 field auditors and 2 staff covering administration and logistics) and is responsible for auditing each of the over 600 suppliers to their company; a responsibility they take very seriously because ‘when you manufacture, you have to be absolutely certain about the ingredients.’ They described the extensive quality control and quality assurance audits they must comply with. These include the Vendor Qualification document covering information on the chemistry of the materials, triennial quality control batch testing, document review, performance testing, proper packaging and mode of transportation, proper labelling with product details like the quantity, storage conditions, handling precautions, manufacturer’s details for traceability and product name; and quality assurance auditing which requires them to go to the supplier and check ‘every activity, every system, every person’.

Extensive regulatory guidelines have established processes and procedures for compliance that are evidenced through documentation. However, we question the assumption that this documentation always provides good evidence of compliance. During the interview, we witnessed a phone call from a supplier to the company whom they had been trying to audit the previous week. The auditees had denied access to some areas of their facility leaving the audit incomplete. The caller said that the paperwork was now all in order so they would just send it without requiring a further site inspection. Our interviewee insisted that they must be permitted to do a full site inspection. After the call ended, our interviewee observed that it would be extremely easy for some companies to just accept the paperwork and not to insist on going back for a full inspection. This left us asking: if the paperwork is in order, is that all that matters?

## Marginal costs and gains

A dominant theme in all our interviews was the costs of doing business. While manufacturers want to make affordable products for consumers/patients and be part of the value chain, they also need to ensure their own survival by making a marginal profit. Companies that fail to do this can rapidly go under in a very tough and competitive market. One company director told us that 90% of the companies that started out at the same time as him had since folded (Interview C).

The need for profit drove almost all the decisions the companies were making. For example, the choice to manufacture intermediates rather than finished formulations was based on the higher costs and risks of formulation production and the difficulties of penetrating the market with a new brand. One interviewee noted that many companies that manufacture formulations engage in ‘contract manufacturing’, that is, making formulations for someone else because market penetration is so difficult (Interview B). Our interviewees estimated the cost of setting up an intermediary-producing company in India was at least US$3 million and required one hectare of space and 20 reactors. After the initial investment the lifetime of pharmaceutical manufacturing equipment is approximately 40 years, but it will start to deteriorate after 7 years because of the corrosive effect of the chemicals. At this point a company will either sell up or reinvest in new equipment (Interview C).

Owners of companies are responsible for the quality of their product but as with any manufacturing enterprise, there are clear incentives to maximise profits. Thus, ‘when a company director is directly involved in production decisions, there is a temptation to cut costs’ (Interview A). One significant production and cost decision is how close to get to the precise dose of API required for a particular medicine. The legal minimum is 95% and achieving higher degrees of purity carries escalating marginal costs. We were told that while some companies will set their own exacting limits of not less than 99% API others will ‘release a batch at 95%’ (Interview A) which, it was noted, could compromise the efficacy and stability of the finished product.

## Establishing trust over complex supply chains

Similar financial pressures drive API manufacturers in India to source raw materials and various stages of intermediates from multiple suppliers in China and other countries. While this helps to keep costs low in some parts of the manufacturing process, arranging procurement and ensuring supplier compliance in the production, packaging and transportation of raw materials across borders present significant challenges. Manufacturers need to either outsource procurement, quality control and quality assurance auditing to local companies or have their own employees based in the supplier countries.

Outsourcing means finding trustworthy partners. One CEO of a small-to-medium-sized company told us that, when he started out, he made some serious mistakes in collaborating with intermediaries who were either incompetent or with whom there were communication difficulties in negotiating prices. He now has two employees whose role is to identify and verify potential suppliers online. He gave an example of the company investing considerable resources doing due diligence on a potential supplier in China by ‘looking at the track record of the company, at their capabilities, doing a market survey, sending employees to meet with the company and using Chinese contacts and consultant’ (Interview B). Contacts are also made at conferences and exhibitions and through personal connections. However, once contracts have been signed, the requirement for ongoing auditing for quality and good manufacturing practice becomes a key component of their relationship.

## Market forces

Our research has highlighted some of the challenges that face API manufacturers in India and has pointed to the potential vulnerabilities for medicine quality that may ensue. The pharmaceutical manufacturing market in India is overcrowded, with a high attrition rate, increasing the pressure to cut costs. The challenges are particularly pressing for small-and-medium-sized enterprises (SMEs) who, as the Office for Economic Co-Operation and Development has noted, typically incur higher costs for ensuring regulatory compliance than larger companies.^25^

One way of achieving financial viability is to outsource several stages of the process to other companies, either within India or overseas (most often to China). As a result, a single API manufacturer in India can deal with several hundreds of suppliers of raw materials and intermediates, with products moving back and forth across borders and jurisdictions many times before the API is complete (even before formulation of the finished product). Trying to keep track of all these partners and ensure that regulatory compliance is achieved at each stage is an expensive and time-consuming process. Inevitably, there are temptations to cut costs, either by circumventing some of the checks or going for minimal regulatory compliance (for example, 95% rather than 99% API).

Other market considerations may be at play and need further investigation. For example, if an assembling manufacturer requires APIs within time-limited windows they may face constraints imposed by monopolistic/oligopolistic supplier(s), willing to supply only a lower grade input in that window of time, driving the price up and increasing the cost for the assembling manufacturer. This, in turn, is likely to lead the assembling manufacturer to not only accept the lower grade input, but also reduce the quality of their output that they supply to the next party in the supply chain to cut costs. In other words, the specificity of windows of time when inputs are required by an assembling manufacturer, may result in them contracting with monopolistic suppliers, who have a ‘take it, or leave it’ attitude—leading eventually to the natural, absorption of lower grade inputs.

The effects of all of this are not fully known but, in combination with cost-cutting mechanisms in other parts of the supply chain such (such as poorer quality packaging), the overall impact on medicine quality and durability could be significant, despite technical compliance at each step of the chain. This presents a significant challenge as India continues to grapple with weaknesses in pharmaceutical regulation and variable standards of the quality of Indian medicines.^26–28^

## Conclusion

Today’s pharmaceutical supply chains are global. Our study has highlighted the complexity and opacity of the crucial steps of API production, where raw ingredients and intermediates cross multiple international borders and jurisdictions before formulation into the final product which itself will be exported internationally. Attending to this early part of the supply chain, and the potential risks arising from regulatory circumvention or minimal compliance, is crucial for ensuring quality further down the line.

From a regulatory point of view, the challenges are substantial. The dispersed and fragmented nature of supply chains contributes to an overall sectorial myopia where each part of the production chain only sees its own section, and no-one has true knowledge and oversight of the whole. It is effectively impossible for any single regulator to keep pace with the pressures of globalisation, underlining the need for increased harmonisation and co-operation to ensure adherence to international pharmaceutical standards.^29^ India’s position as ‘pharmacy to the world’ depends very substantially on many SMEs who manufacture, import and export raw ingredients, intermediates, APIs and finished products. These companies typically operate on small margins, so any measures to improve effective compliance must be managed carefully, to ensure the continued viability of the sector.

This work is based on interviews with a small number of manufacturers and regulators in India and relies on their understandings of the problem and their willingness to be candid with the interview team. Manufacturers have much to lose in terms of reputation. Some interviewees were clearly concerned about ‘saying too much’, with many preferring to speak in general terms about the issues they faced rather than giving specific examples. Moreover, with a small sample size, we cannot draw definitive inferences about the wider pharmaceutical sector in India, or in other countries. Nonetheless, this study highlights the vulnerabilities within the initial stages of pharmaceutical manufacture that have hitherto been largely overlooked by researchers. Investigating these issues more thoroughly and across multiple contexts will be a crucial step in eliminating SF medical products from global supply chains, given that the quality of a finished product can only ever be as good as the quality of its API.

## Data Availability

Data is available upon reasonable request. Interested persons should contact the corresponding author.
