# Supplementary material for: Insights from a qualitative study of the procurement and manufacture of active pharmaceutical ingredients in India
Source: BMJ Glob Health. 2023 May 17;6(Suppl 3):e011588. doi: 10.1136/bmjgh-2022-011588 (PMC10201222; doi:10.1136/bmjgh-2022-011588)
Supplement: Supplementary data [file bmjgh-2022-011588supp001.pdf]

Appendix S1 – Reflexivity Statement

1. How does this study address local research and policy priorities?

Poor quality medicines are a major threat to healthcare provision in low and middle-income countries. The problem exacerbates disease vulnerabilities of already disadvantaged populations including children, women and the elderly. However, while the higher-level structural drivers of this problem are well established, little is known about the operation of complex supply chains for the procurement of raw materials and the manufacture of APIs that are then used to formulate branded or generic pharmaceuticals and their vulnerability to penetration by poor quality products.

2. **How were local researchers involved in study design?**

Two of the authors (PM and HV) originate from, and resided (at the time of the research) in India. PM is a senior academic research leader who was involved in the design of the project, and facilitated the data collection. He was also involved in manuscript preparation. HV was not involved in the design stage but was an interviewer and offered key insights in the data analysis. In addition, there were high-income country researchers with extensive experience of conducting, leading, or organising international research collaborations involving low- and middle-income countries (HH and KH).

3. How has funding been used to support the local research team?

This project was with a very experienced and skilful local research team. It enabled them to develop their research and analytical skills working in a new empirical context and setting with which they were previously unfamiliar.

4. How are research staff who conducted data collection acknowledged?

All research staff in who took part in the research design, collection and analysis of data are included as authors.

5. Do all members of the research partnership have access to study data?

All members of the partnership have access to data.

6. How was data used to develop analytical skills within the partnership?

The analytical skills of the local research team were enhanced by being actively engaged in the process of iterative research moving from data to theory and back again throughout the data collection period. As the interviews were conducted, the research team were constantly engaged in conversations and discussions about the content of the interviews and how they might be analysed and understood.

7. How have research partners collaborated in interpreting study data?

See answer to q 6.

8. How were research partners supported to develop writing skills?

The research team writing this paper is composed of three senior and one junior researcher. The early career researcher (HV) on the authorship team will be supported by senior colleagues to develop and refine their writing skills through the drafting and revision of this paper.

9. How will research products be shared to address local needs?

This paper will be published as open access. We have developed a post-publication dissemination plan in consultation to distribute recommendations across a wide constituency. This will include engagement with research leaders in global health and other fields involved in international collaborations and with academics and policy makers in based in both high-income countries and low- and middle-income countries.

10. How is the leadership, contribution and ownership of this work by LMIC researchers recognised within the authorship?

Authors HV and PM worked to develop and write this manuscript, and their contribution has been recognised as by the author order.

11. **How have early career researchers across the partnership been included within the authorship team?**
HV is an early career researcher and recognised as an author in this paper.

12. How has gender balance been addressed within the authorship?

Three authors are female (HH, KH AND HV) and one author is male (PM).

13. How has the project contributed to training of LMIC researchers?

HV developed her interviewing and analytical skills during this project and was subsequently supported in her successful PhD application by PM and KH.

14. How has the project contributed to improvements in local infrastructure?

This project has not directly contributed to improvements in local infrastructure.

15. What safeguarding procedures were used to protect local study participants and researchers?

Safeguarding issues were directly addressed through the ethics approval given by the IRBs at Durham University UK, Oxford University UK and Ashoka University, UK
